# The clinical effect of an unloader brace on patients with osteoarthritis of the knee, a randomized placebo controlled trial with one year follow up

**DOI:** 10.1186/s12891-018-2256-7

**Published:** 2018-09-22

**Authors:** Hjörtur F. Hjartarson, Sören Toksvig-Larsen

**Affiliations:** 1 Dept of Orthopedics, Landspitali University Hospital, E-4 Fossvogur, 101, Reykjavik, Iceland; 2 Dept of Orthopedics, Hässleholm hospital, Esplanadgatan 19, 281 38 Hässleholm, Sweden; 3 Lund University, Lund, Sweden

## Abstract

**Background:**

Treatment of patients with knee osteoarthritis is challenging. Unloader braces have been developed with various success. Unloader One® Knee Brace is light, easily-fitted and shown to be effective by the unloading of the affected compartment. The aim of the study was to assess the clinical outcome of the brace vs. a placebo on patients with knee osteoarthritis.

**Methods:**

Initially 150 patients were randomized to receive either the Unloader brace or a control placebo group look-alike brace where the active strips had been removed. The patients were followed up at 6,12,26 and 52 weeks with Knee Society Score (KSS) and Knee injury and Osteoarthritis Outcome Score (KOOS). The reason for dropout was recorded.

**Results:**

A total of 149 patients were included, 74 in the study and 75 in the control group. The mean age was 59.6 vs. 60.2, BMI was 27.5 vs. 26.9, 37% vs. 44% were women in the study vs. control group. Both groups showed improvement in KSS over 52 weeks, with the study group showing higher improvement in mean scores. KSS increased from 64.3 to 84.0 for the study group and from 64.0 to 74.6 for the control group (*p* = 0.009). The study group improved in KSS function from 67.0 to 78.6 (*p* < 0.001) and KOOS for knee related symptoms increased/improved from 64.3 to 72.4 (p < 0.001). Activity of daily living increased/improved from 65.3 to 75.2 and Sports/Recreation from 24.6 to 40.2 (*p* > 0.001) whereas the control group did not show significant improvements in any of the scores. The dropout was higher in the control group, 40 vs. 25.

**Conclusions:**

The brace seems to be more effective and better tolerated than the placebo.

**Trial registration:**

The trial was retrospectively registered with ClinicalTrials.gov (NCT03454776) on March 6th 2018.

## Background

Osteoarthritis (OA) is a progressive degenerative joint disease that involves damage to the joint cartilage and changes in the subchondral bone and connective tissue of the joints [1]. Risk factors include obesity, occupation, trauma, excess joint load and hereditary factors [1, 2]. As populations age and obesity increases the burden of knee OA is rising [3]. No cure exists for OA and all current treatments focus on symptom alleviation. Even though there are numerous well-documented treatments available, the treatment of OA of the knee in younger patients as well as older patients with mild to moderate pain still poses a challenge. The current consensus is that non-operative treatment is recommended before surgery, with a combination of pharmacological treatment and non-pharmacological modalities, such as patient education, physical therapy, weight loss, walking aids and braces [4]. In unicompartmental knee OA with valgus/varus misalignment, unloading of the affected compartment has shown to be effective in biomechanical studies [5]. Knee braces unloading the diseased compartment have been shown to be effective in several studies and are included in many guidelines for the treatment of symptomatic knee OA [6–9]. Many of these studies are limited by having few test subjects or short follow up times. Some studies have failed to show benefit of braces compared to other treatments [10] and a Cochrane review [11] published 2015 states that evidence for the use of braces is inconclusive. In this review osteoarthritis of different types and severities are analyzed. In this study only patients with mild to moderate osteoarthritis (Ahlbäck grade I-II) were included, as we believe this to be the target group for this type of treatment. The aim of this study was to assess the one-year clinical effect of an unloader knee brace compared to a placebo, evaluate compliance and reasons for discontinuing treatment.

## Methods

This study was a randomized placebo controlled study in patients with mild to moderate knee OA. All patients included were initially treated in primary care settings, with patient education, physical therapy and analgesic use. Patients visiting our outpatient clinic who met the inclusion criteria were asked to join the study. Some patients were recruited after responding to advertisements in local newspapers and social media. The first patient was included in April 2012 and the last in August 2014. The follow up time was 12 months. An orthopedic surgeon evaluated symptoms and radiographic evidence before inclusion in the study. All patients between 30 to 70 years of age, with knee pain for more than three months, with arthroscopic or radiographic evidence of knee OA (Allbäck or Kellgren-Lawrence grade 1–2) [12], and with BMI < 35 were eligible for the study. Patients who had prior major surgery to the same knee, a history of stroke, neurological or psychiatric problems, patients using opioids or steroids, as well as patients with rheumatoid arthritis, immunological depression or other severe medical problems were excluded.

**Fig. 1:**
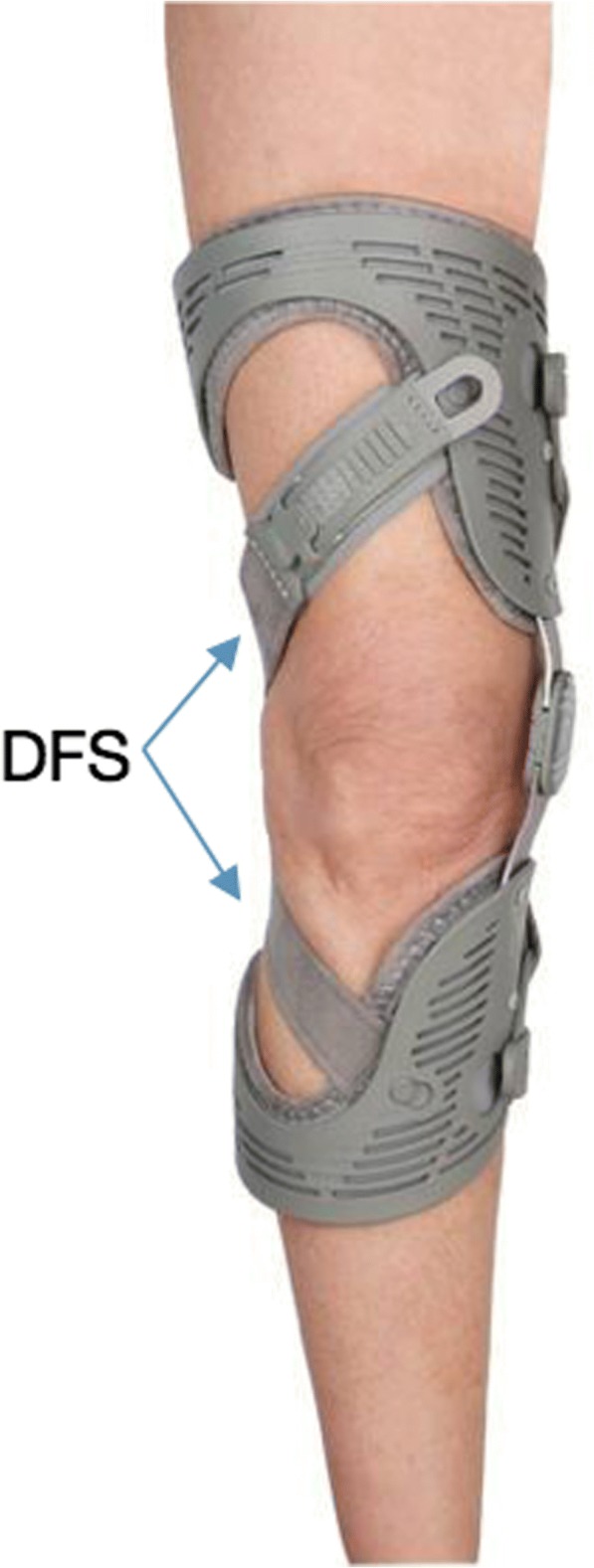
Unloader One® brace, Ossur, Iceland

150 patients were randomized to either a study group or a control group and followed for one year. The study group received an Unloader One® knee brace (Ossur, Iceland, Fig. 1). The Unloader One uses two Dynamic Force Straps (DFS) to impart a force against the lateral side of the knee as the knee extends. The purpose of this force is to reduce the load in the medial knee compartment. The control group received an Unloader One brace, with the DFS removed. The purpose was to create a device that looked like the Unloader One® brace but without its functionality.

Information about age, gender, height, weight and occupation were collected. A study nurse randomized the patients to either of the two groups. An orthopedic technician fitted all the braces and the researchers collecting all the data were blinded. All patients were graded by a physician for Knee Society Score (KSS) [13] and answered self-administered questionnaires; Knee injury and Osteoarthritis Outcome Score (KOOS) [14] before applying the brace at baseline and after 6,12,26 and 52 weeks. The KSS is a mixed outcome score, which is both objective and subjective. The KOOS is a self-administered patient reported outcome measurement questionnaire divided into five subcategories of pain, knee related symptoms, activities of daily living (ADL), sport and recreation (S&R) and quality of life (QoL). When answering the KOOS, patients are asked to consider their experience over the previous week. The results from both of these measurements are presented on a best-to-worst scale from 100 to 0. The KSS Score and function as well as the five subgroups of KOOS were determined as primary endpoints of the study. The reasons for dropout were documented, as well as analgesic use, frequency of doctor visits, absence from work and changes in employment status, which were considered secondary endpoints. Initially an evaluation of the cost of treatment and economic aspects were included as a secondary outcome, but were later discarded due to lack of data. All the patients from the control group that dropped out of the study due to ineffectiveness or mechanical problems were informed that they had a placebo brace and were offered to try out the original brace as one of the treatment options available to them. The treatment is not considered harmful but the placebo treatment may cause delay in treatment.

The data was analyzed and 95% confidence interval was calculated using a mixed model repeated measures analysis of variance. This method uses all available information and includes justifications for baseline imbalance with stratification of randomized confounding factors. An independent statistician analyzed all data presented. Statistical analysis was performed with STATA (StataCorp. 2015. Stata Statistical Software: Release 14. College Station, TX: StataCorp LP.) Power and sample size calculation was done with the PS: Power and Sample Size Calculation program by William D. Dupont and Walton D. Plummer, Jr. version 3,0. 2009 [15].

The results of data collected from KSS and KOOS are presented as estimated marginal mean difference at 6, 12, 24 and 52 weeks and 95% confidence interval.

## Results

**Fig. 2:**
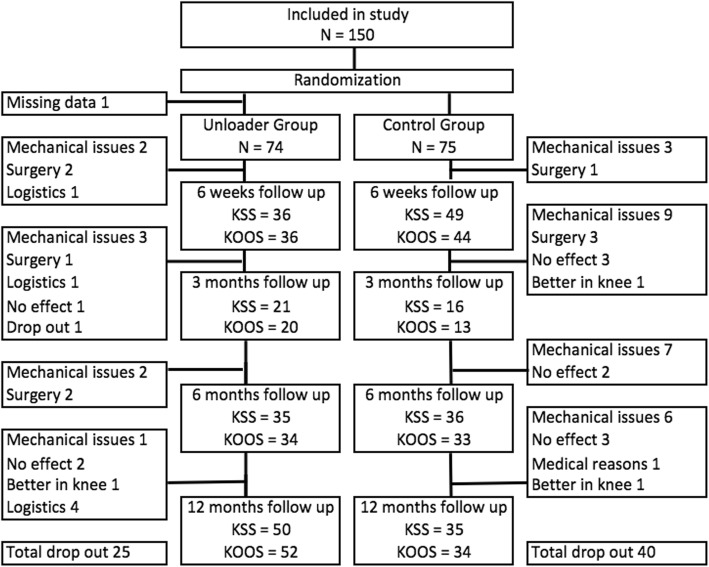
Participant flow. KSS and KOOS show how many participants were evaluated for KSS or turned in KOOS questionnaires at that follow-up

In total 149 patients answered questionnaires at baseline, 74 in the study group and 75 in the control group. All patients had unilateral medial OA, 80 in the left knee and 68 in the right knee. After 52 weeks 85 patients were still participating in the study, 50 in the study group and 35 in the control group. The reasons for dropout are shown in the flowchart below (Fig. 2). The main reasons for dropout were mechanical problems while using the brace. These problems include problems using the brace while working, sliding off, rubbing, feeling of instability and some patients found the brace to cumbersome to use. Nine patients underwent surgery before the study time was completed, 5 in the study group (3 total knee replacements, 1 total hip replacement, 1 high tibia osteotomy) and 4 in the control group (2 total knee replacements, 1 high tibia osteotomy and 1 spinal surgery). Some patients chose to discontinue the study due to work or lack of time, but continued to use the unloader brace, and are listed under logistics. Three patients reported silicone reaction or allergy as one of the reason for stop using the brace. One patient was included and given a subject number, but for reasons unknown to us, his data is missing and no record of him being fitted with a brace or called for evaluation. This explains why there are only 149 patients in the study.

### Demographics

The baseline demographic and clinical characteristics are shown in Table 1.

**Table 1:** Baseline demographic and clinical characteristics

Baseline characteristics	Study Group	Control Group
Participants	74	75
Age, in years (mean, SD)	59.6 (8.0)	60.3 (6.9)
Female gender	27 (37%)	33 (44%)
BMI (mean, SD)	27.5 (3.0)	26 (3.0)
KOOS Pain (mean, 95% CI)	61.2 (58.7–63.7)	61.1 (58.7–63.6)
KOOS Symtom (mean, 95% CI)	64.6 (61.8–67.2)	64.4 (61.7–67.1)
KOOS ADL (mean, 95% CI)	65.3 (62.9–67.7)	65.1 (62.7–67.5)
KOOS Sport/Rec (mean, 95% CI)	14.6 (21.6–27.6)	25.0 (22.0–28.0)
KOOS QoL (mean, 95% CI)	52.2 (49.4–55.0)	52.3 (49.5–55.1)
KSS Score (mean, 95% CI)	64.3 (60.3–67.6)	64.0 (60.3–67.6)
KSS Function (mean, 95% CI)	67.0 (64.0–70.1)	67.1 (64.0–70.3)

### Primary endpoints

#### KSS

**Fig. 3:**
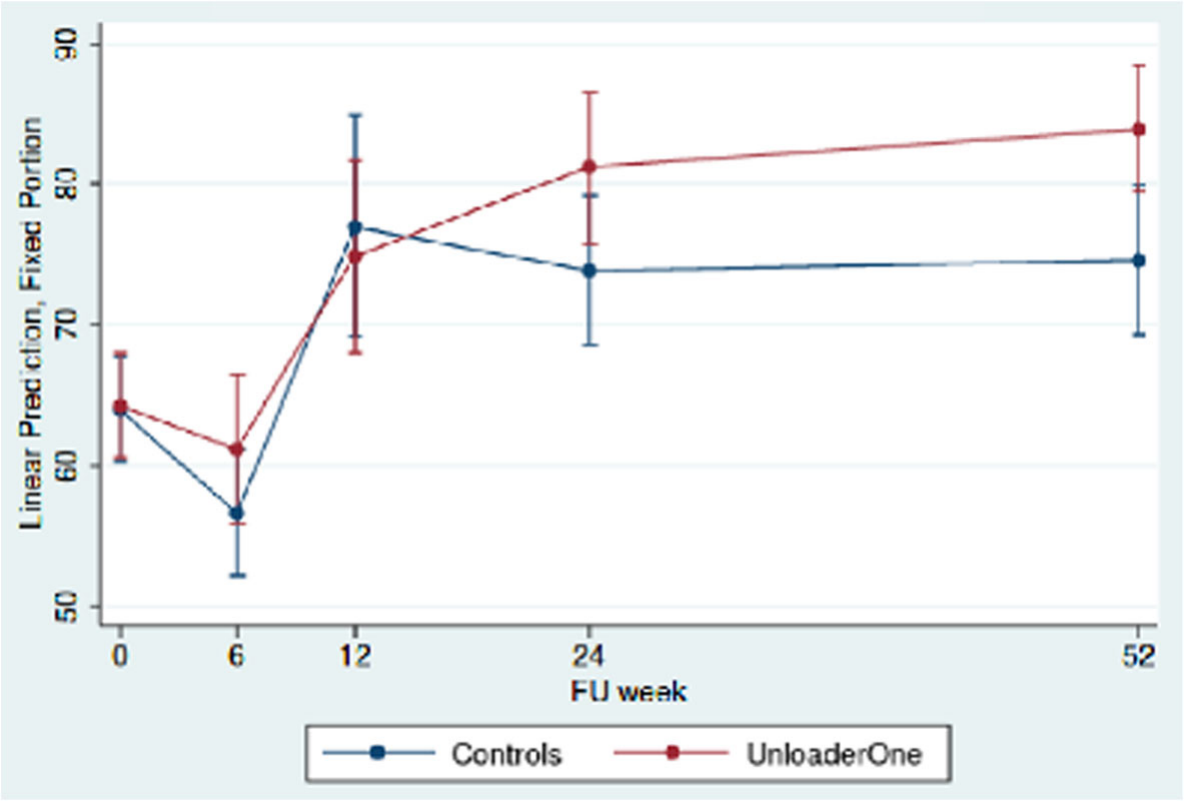
Predictive margins of KSS with 95% CI at baseline 6,12,24 and 52 weeks

Both groups showed an initial decline in KSS at six weeks indicating worsening of symptoms, followed by increase at three months indicating fewer symptoms, which continued until the end of study at twelve months. At six months the study group showed more improvements than the control group and the difference had increased even further at twelve months from 64.3 (95% CI 60.6–68.0) to 84.0 (95% CI 79.5–88.5) compared to 64.0 (95% CI 60.3–67.6) to 74.6 (95% CI 69.3–80.0). The difference at twelve months between the two groups is 9.4 (95% CI 2.4–16.4). (Fig. 3).

#### KSS function

**Fig. 4:**
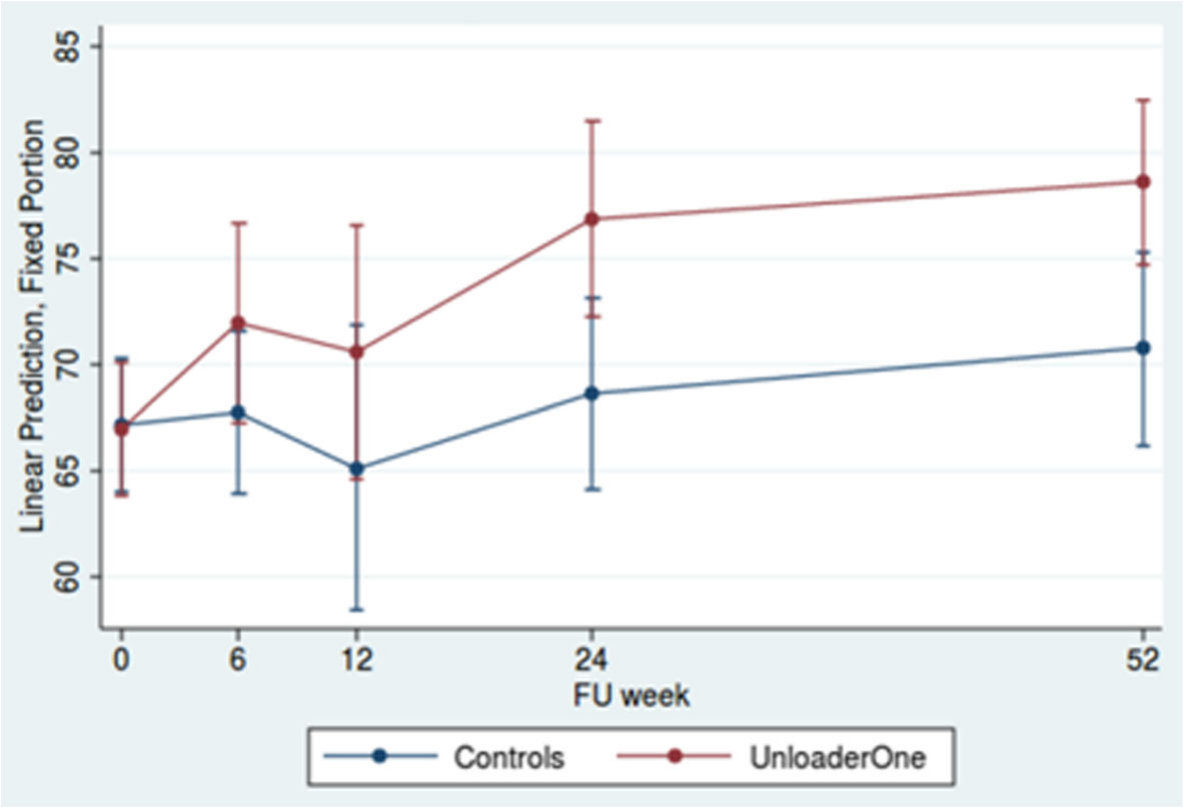
Predictive margins of KSS Function with 95% CI at 6,12,24 and 52 weeks

The study group showed improved function at six weeks, as measured with the KSS functional measurement, with further improvements at 12, 24 and 52 weeks. The study group improved from 67.0 (95% CI 64.0–70.1) to 78.6 (95% CI 74.7–82.5) at 52 weeks, with a difference of 10.6 (95% CI 4.1–17.1). The control group showed slight improvement from 67.1 (95% CI 64.0–70.3) to 70.8 (95% CI 66.2–75.3) with the difference between the groups 7.8 (1.9–13.8) (Fig. 4).

#### KOOS

The adjusted mean values of the KOOS are listed in Table 2.

**Table 2:** KOOS change from baseline to week 52, adjusted mean values and 95% confidence intervals

Outcomes	At baseline		At 52 follow up		Change from baseline		Between group
	Unloader	Controls	Unloader	Controls	Unloader	Controls	difference
KOOS Pain	61.2 (58.7, 63.7)	61.1 (58.7, 63.6)	68.9 (66.0, 71.8)	63.7 (60.2, 67.3)	7.7 (4.2, 11.2)	2.6 (1.4, 6.6)	5.2 (0.6, 9.8)
KOOS Symptom	64.5 (61.8, 67.2)	64.4 (61.7, 67.1)	72.4 (69.2, 75.6)	65.4 (61.6, 69.3)	7.9 (4.0, 11.8)	1.0 (−3.4, 5.5)	6.9 (1.9, 11.9)
KOOS ADL	65.3 (62.9, 67.7)	65.1 (62.7, 67.5)	75.2 (72.4, 78.0)	66.9 (63.5, 70.3)	9.8 (6.4, 13.3)	1.8 (−2.1, 5.8)	8.2 (3.8, 12.6)
KOOS Sport/Rec	24.6 (21.6, 27.6)	25.0 (22.0, 28.0)	40.2 (36.7, 43.8)	27.8 (23.4, 32.1)	15.7 (11.3, 20.0)	2.8 (−2.2, 7.8)	12.5 (6.8, 18.1)
KOOS QOL	52.2 (49.4, 55.0)	52.3 (49.5, 55.1)	55.7 (52.4, 59.0)	49.5 (45.4, 53.6)	3.5 (−0.6, 7.6)	−2.7 (−7.5, 2.0)	6.1 (0.9, 11.4)

##### KOOS pain

On the pain subscale of KOOS, the study group improved from 61.2 (95% CI 58.7–63.7) to 68.9 (95% 66.0–71.8) at 52 weeks, with a difference of 7.7 where a difference of 10 is considered a clinically detectable difference. The control group showed less improvement with a difference between the groups of 5.2 (*p* = 0.02). This difference is not considered clinically important.

##### KOOS symptoms

The study group improved from 64.5 (95% CI 61.8–67.2) to 72.4 (95% CI 74.7–82.5) whereas the control group shows hardly any improvement from baseline. The difference at 52 weeks from baseline for the study group is 7.9 which is slightly less than 10, the clinically detectable cut off mark traditionally used in KOOS scores.

##### KOOS activities of daily living

The sub score for ADL shows an increase in the study group from 65.3 (95% CI 62.9–67.7) to 75.2 (95% CI 72.4–78.0) and a difference of 9.8 (*p* < 0.001) while the control group shows no statistically significant difference from baseline.

##### KOOS sports and recreation

The sub score for sport and recreation increased for the study group during the whole follow-up. The scores for the study group increased from 24.6 (95% CI 21.6–27.6) to 40.2 (95% CI 36.7–43.8) with a difference of 15.7 (*p* < 0.001). The control group did not show much improvement during this time and the difference between the groups at 52 weeks was 12.5 (p < 0.001), which is both a clinical and statistical significant difference.

##### KOOS quality of life

The sub score for quality of life did not show much change for either group, the study group increasing from 52.2 (95% CI 49.4–55.0) to 55.7 (95%52.5–59.0) and the control group actually decreasing from 52.3 (95% CI 49.5–55.1) to 49.5 (95% CI 45.4–53.6). The difference between the groups at 52 weeks was 6.1 (*p* = 0.02) which is not a clinically detectable difference.

## Discussions

This exploratory study indicates that pain and function (activity of daily living and sports and recreation) can be improved in comparison to placebo during one-year use of the Unloader brace. The study showed some improvements in primary endpoints except in the KOOS subgroup measuring quality of life at one year follow up. The differences between the groups at 6, 12 and 24 weeks are hard to interpret due to poor return at these intervals. Most patients showed up at some of the follow up appointments i.e. either at six weeks or three months but not both, and some showed up at all appointments. Some subjects stated that they did not have time for all the follow-up visits. Some patients choose not to come to the appointment but sent in the self-administered questionnaires, and others showed up at the appointments but neglected to send in the self-reported questionnaires.

There were more dropouts from the control group than the study group, mostly due to mechanical issues, such as the brace sliding of the leg or hurting the patient.

It is interesting to note that improvements in KSS score are not evident at the six week follow up and the difference between groups does not become apparent until after six months suggesting that long term follow up is needed to see the difference. Many studies are limited by short follow up times. This also suggests that it may take time for the patients to adjust to the brace in the clinical setting and full results should not be expected immediately.

The high dropout rate in the control group compared to the study group shows in our opinion that the design of the brace matters and supports the theory that unloading the affected compartment can relief pain and improve function. With this said, it should be kept in mind that the placebo braces might still have clinical effect as shown in biomechanical studies even though the dynamic tension strap has been removed. This effect might be due to diminished muscular co contractions rather than compartmental unloading as suggested by Ramsey et al. [16].

The KSS questions for pain and function ask patients to remember their experience the previous four weeks and the KOOS questionnaire the previous week. It is a thought for mind if these kinds of outcome scores are appropriate for measuring outcomes for a device that is only used during activity rather than continuous pain relieving effect such as medication or operations. This might explain larger differences between groups in functional scores rather than pain and symptom scores.

There is a clinically and statistically significant improvement in KSS and KSS function for both groups at one year compared with baseline. These results are compatible with previously published studies [17, 18]. There is also difference between groups but it is not statistically significant. For KOOS Pain, symptoms and quality of life there is a slight improvement at one year compared with the baseline for the control group even though it’s questionable that this improvement is clinically significant. There is both a clinical and statistical significant improvement in the category of sport and recreation in the study group, with significant difference between the groups at one year follow up.

In the study protocol we had set the economic aspects of brace use as the secondary endpoint. This proved to be more complex than we anticipated and beyond the scope of this article and therefore the secondary endpoint was limited to the dropout rate.

## Conclusions

The results of this study reflect our experience in clinical practice, there are many who find braces useful and there are those who don’t think that they add anything to their treatment. Further research should be focused on identifying the responders and non-responders of this treatment applying OARSI criteria [19] for responders and to see what if any differentiates these groups.

## Abbreviations

ADL: Activities of daily living
BMI: Body max index
KOOS: Knee and Osteoarthritis Outcome Score
KSS: Knee Society Score
OA: Osteoarthritis
OARSI: Osteoarthritis Research Society International
QoL: Quality of life
S&R: Sports and recreation
